# The Treatment of Glycosuria

**Published:** 1889-05

**Authors:** Charles W. Purdy

**Affiliations:** Chicago, Ill.


### THE TREATMENT OF GLYCOSURIA.

By CHARLES W. PURDY, M. D., Chicago.

It is customary to consider glycosuria under two forms: First, a milder manifestation of the disease in which only small amounts of sugar appear in the urine, and these often intermittently while tbe general health of the patient suffers little or no disturbance. Second, a more severe type of the disease characterized by excessively saccharine urine, great thirst, polyuria, emaciation, etc., leading more or less rapidly to extreme marasmus and death. The first form is chiefly of reflex origin, and hence its milder type and rarely fatal termination; while the second form is doubtless of central origin, and consequently more pronounced and serious in its consequences. In a systematic consideration of the management of glycosuria it is important that these two types of the malady be constantly kept in mind.

Physiological chemistry has shown us that glycosuria expresses itself chiefly through disturbance of the glycogenic function of the liver. Claude Bernard extended our knowledge a step farther, and showed that the elemental cause consists of some disturbance of the central nervous system, closely corresponding to the vaso-motor centre. All attempts, however, to unravel the nature of this disturbance through the aid of morbid anatomy have proved thus far entirely futile. It is well to remember, however, that in careful scientific research, failure often teaches us valuable lessons, and, indeed, often furnishes useful information. The very fact that the study of morbid anatomy in glycosuria has failed to reveal uniform and tangible lesions of the central nervous system, goes far to form a presumption that if lesions exist in these cases they can scarcely be sufficienly grave in themselves to cause fatal results. Our present knowledge of the nature and course of glycosuria is quite in harmony with this presumption; for indeed we find the cause of death uniformly to depend upon the perverted function of organs widely apart from the brain. Moreover, if the perverted function of these organs can be corrected and held under control the patient may survive almost indefinitely.

Without entering into the discussion of the many theoretical questions with which, unfortunately, our knowledge of glycosuria is at present so deeply involved, let us more practically inquire, what facts have we at command upon which to base a rational system of managing the disease? We know that the chief expression of glycosuria is a perverted elaboration of the hydro-carbon foods in the liver, resulting in their conversion into grape sugar. We know that the surcharging of the blood with large quantities of this sugar, not only gravely alters the nutritive qualities of the blood, but it is also liable to induce chemico-toxic changes in that fluid, which are dangerous to life. We know, in short, that the perverted elaboration of so large a proportion of the food supply as that of the hydro-carbonaceous, the saturation of the tissues with the resulting morbid products, and the necessary efforts at their elimination, lead to altered nutrition, emaciation, wasting of the vital forces of economy, secondary disease of important organs, and to that complex of morbid processes that in glycosuria bring about exhaustion and death. Now, obviously, if we can succeed in cutting off completely the supply of such foods as are prone to faulty elaboration—for the most part the hydro-carbons—we shall not only arrest the perverted liver function, but we shall also save the system from damaging effects of the morbid products poured into it through faulty elaboration of food, and thus practically arrest the regressive changes that lead to such grave results.

If we had to deal only with the purely hydro-carbon foods as the exlusive source of sugar production in the economy, our problem would be a comparatively simple one; since a thoroughly nourishing and sustaining diet can be furnished exclusive of these. But while the hydro-carbons are the chief, they are not always the only source of sugar production. Experimental investigation has shown that when animals were fed on purely nitrogenous foods—even for lengthy periods of time—a small amount of glycogen still continued to be present in their livers. In the most grave forms of diabetes the "sugar-forming vice" of the organism becomes so strong that the liver seems capable of splitting up a portion of the nitrogenous foods, and even of the albumenoids of the tissues, and of transforming a part of these into sugar. Fortunately such cases are for the most part long-neglected or advanced ones. Although much may be accomplished even here in retarding the disease, yet it may, as a rule, be considered progressive towards a fatal termination.

The sugar-forming powers of the organism in glycosuria are feeblest in their operation upon nitrogenous materials; indeed in the early stages of the disease it is probable that these always escape sugar transformation, and of this class starch is the most dangerous element.

Practically then the more completely we are able to eliminate the hydro-carbons from the food supply in glycosuria, the more completely will we be able to bring and to hold the disease under control. Certain allowances must be made for individual idiosyncracies, as well as for a few exceptional articles of diet, which experience has shown us are sometimes well borne—even when their classification would seem to contra-indicate their use. To speak more accurately then, the more completely we are able to supply the system with that which it can appropriate as nourishment, and at the same time the more completely we can eliminate that which is convertible into sugar, the more successful will be the treatment. Now, in view of the above facts, which I have endeavored to present as carefully separated from theoretica speculations as possible, it seems indeed strange that more earnest efforts are not made in the management of glycosuria—especially in the more pronounced types of the disease—to supply more nearly that diet upon which almost alone depends the improvement or cure of these cases. I shall first point out what seem to me the more prominent errors commonly made in dieting in the severe type of the disease, giving a list of the admissible foods; after which I shall note some of the liberties of diet that may be indulged in the milder reflex forms; and lastly, I shall refer to the influence of drugs over the disease.

First in importance comes the question of bread, some form of which containing starch is permitted in all the diet lists I have seen. Now I do not hesitate to state, without fear of successful contradiction, that all the so-called diebetic flours, breads, and cakes in the market are loaded with hydro-carbons. They are "a snare and a delusion," and have unquestionably shortened the lives of thousands. Most samples of gluten flour, from which the starch is claimed to have been eliminated—or nearly so—contained from 20 to 40 per cent. of starch. I saw in Dr. Pavy's laboratory in London, a few months since, an analysis of one of the so-called diabetic flours on sale in our markets, which showed the starch contents to be nearly 60 per cent. Long before I became aware of these facts I found that I could not control typical cases of diabetes if I permitted the use of commercial flours so-called *diabetic*. I need scarcely add that with the above figures before me I have discarded them altogether.

The withdrawal of bread from the diet usually constitutes the most serious deprivation the diabetic patient has to encounter, although the appetite for bread is more largely a matter of taste and habit than of necessity. Some patients become quite reconciled to the change after a few weeks, and do not mind it, but usually the craving for bread of some kind remains more or less strong, and will not be supplanted by the use of other foods. In the latter class of cases, if strict dieting be demanded, I permit the moderate use of bread made from almond flour as first practiced, I believe, by Dr. Pavy. The almond is absolutely free from starch, and contains about 6 per cent. of sugar. The latter may be eliminated by boiling the meal in acidulated water for an hour or so and then straining it. The almond meal is not on sale in the markets; the large percentage of its contained oil (50 per cent.) renders it unfit for keeping sufficiently long for commercial purposes. In my own practice I direct the meal to be made as required by means of mills especially constructed for the purpose. Almond flour, when beaten up with eggs, may be raised with the aid of a little baking powder, and baked in small tins in an oven, and the resulting bread is relished by most of my patients as equally palatable with ordinary bread. It should be borne in mind that almond bread, as indeed all substitutes for common bread, should be used in moderation; otherwise patients deprived of other luxuries of food, fly to the permitted bread with an avidity seemingly born of the thought that it is indeed the "staff of life," instead of merely a substitute therefor. To make a substituted article of diet go further than the original one is more than is to be expected, even in these practical days, and yet I am led to believe that the failure in accomplishing this in the case of almond bread has led to its unjust condemnation by some in these cases.

The next question of importance in diet—and one upon which authorities greatly differ—is the propriety of the use of milk in diabetes. Dr. Donkin, perhaps the most enthusiastic advocate in its favor, published a book in 1871, which was devoted to the exclusive use of milk as a means of treating this disease. In England Dr. Donkin's so-called "milk cure" has met with few, if any, weighty supporters; on the contrary, many advocate the total exclusion of milk from the diet. My own experience in the use of milk in the treatment of diabetes began nine years ago, since which time I have made thorough and varied trials of it, both as an exclusive and as an adjunct diet. My conclusions are that milk is successful chiefly—perhaps only—in milder forms of the disease, such as I have termed reflex cases. Such cases are, as a rule, controllable by moderate limitations of diet, which offer greater range and nutritive power than does milk. In the more severe type of the disease I have repeatedly found, when the diet was rigidly restricted, save in the use of milk, that the total exclusion of the latter without other change caused a prompt reduction, and often the disappearance, of sugar from the urine.

Milk contains a very considerable amount of sugar (lactine), about half an ounce to each pint, and Dr. Pavy observes that this animal hydro-carbon "comports itself in the intestinal canal precisely as does grape-sugar." There can be little doubt, therefore, that in the more prônounced type of diabetes requiring a strict diet, milk should be excluded from the list.

There is a form of glycosuria that occurs in obese and over-nourished subjects, in which the amount of sugar in the urine is usually small, and probably largely due to the ingestion of more hydro-carbons than the system is able to appropriate. Such cases are benefitted, and indeed often cured, by a course of fasting. The "milk cure," consisting of the exclusive use of skimmed milk, is likely to benefit such cases because it is, in fact, a system of starving. Skimmed milk alone is not sufficient to long maintain proper nourishment to the system. In pronounced diabetes of central origin, where the assimilative powers of the system are weakened, and more or less emaciation has set in, it would, therefore, seem absolute folly to confine the patient to skimmed milk, for under such circumstances death from inanition must be but a question of a short time. Sir William Roberts records three cases which he subjected to the "milk cure" with the result that they all succumbed in a short time. My own experience is similar to Dr. Roberts', save that I ceased to use it as an exclusive diet after seeing my first patient rapidly sink under its employment. It is important to bear in mind that lactine is confined to the whey—and consequently the other derivatives of milk, as cheese, cream, curds and butter, are unobjectionable.

Another food of animal source contra-indicated in diabetes is liver. The liver of animals contains considerable sugar, as might be expected, considering the glycogenic function of that organ. Not only should the liver of quadrupeds be avoided, but certain fish, especially oysters and the interior of crabs and lobsters, since they possess proportionately very large livers. It has been claimed that this precaution is more in keeping with theory than practice, but a sufficient answer is furnished in the fact that analyses of oysters have shown as high a range as 10 per cent. of sugar.

The very wide distribution of starch and sugar throughout the vegetable kingdom renders our selection of food from this source limited indeed. In strict dieting, we are obliged to avoid nearly the whole list of table vegetables. One class only are we at all safe in drawing upon—greens—and these with caution. Green vegetables fortunately consist mostly of cellulose and contain little, sometimes no, starch or sugar. They are rendered still safer if boiled before being eaten; the hot water further ensuring the absence of starch and sugar.

The starch and sugar composition of vegetables varies somewhat. This variation depends much upon the degree of cultivation, and the nature and climate of the soil in which they are produced. As a rule, a high degree of domestic cultivation favors an increase of starch and sugar, while high temperature and sunny skies have an opposite tendency. Among the least objectionable vegetables may be mentioned, spinach, lettuce, olives, cucumbers, mushrooms, Brussels, sprouts, turnip-tops, water-cresses, cabbage, cauliflower, and the green ends of asparagus. Nearly all nuts are unobjectionable, chestnuts forming an exception.

In the matter of beverages the diabetic patient will scarcely encounter very serious restrictions, since the range permitted includes most of those in domestic use, including many which fall within the line of luxuries. Among these may be mentioned tea, coffee, all mineral waters, pure spirits, as brandy, whisky, gin, and such wines as claret, Rhine wine and Burgundy.

Having briefly reviewed the food products applicable in glycosuria, I shall now enumerate the list I employ in dieting patients upon strict principles, as appropriate in the more severe type of true diabetes of central origin.

STRICT DIABETIC DIET.—Meats of all kinds except livers, beef roasted, broiled, dried, smoked, cured, potted, or preserved in any way except with honey, sugar, or prohibited vegetables. Mutton, ham, tongue, bacon, sausages. Poultry and game of all kinds. Soups made from meats, without flour or prohibited vegetables. Eggs, butter, cheese, pure cream, curds, oil, gelatine, and unsweetened jellies. Fish of all kinds except oysters and the inner parts of crabs and lobsters. Bread, biscuits, and cakes made from almond flour. Spinach, lettuce, olives, cucumbers, mushrooms, water-cresses, green cabbage. Almonds, walnuts, Brazil nuts, filberts, butternuts, cocoanuts. Salt, vinegar and pepper.

DRINKS.—Tea and coffee, mineral waters, whisky, gin and brandy, in moderation. Claret and Rhine wine.

In mild forms of glycosuria some additions may be safely made to the above diet, and often with advantage. Since in such cases the sugar-forming powers of the organism are weaker; or, in other words, the assimilative powers for sugar and starch are greater, it is only necessary to limit, *not to curtail*, the hydro-carbons. It seems necessary, therefore, to have at hand to draw upon a supplemental list of foods, which contain but limited amount of these agents. The selection from the supplementary list should always be made with care; indeed, it should be almost as much a matter of experiment as a rule, since we encounter wide differences in individual cases. Thus levulose—fruit sugar—is often well assimilated in the milder form of the disease, and this permits the inclusion of certain fruits in the supplementary list.

SUPPLEMENTARY DIET.—Cabbage, celery, radishes, cauliflower, green string beans, cold slaw, kraut, young onions, tomatoes, cranberries, apples, if not sweet, milk in moderate quantities, and bran bread or gluten bread well toasted.

The discovery of saccharin has furnished us an admirable substitute for sugar, since this agent possesses a sweetening power nearly three hundred times greater than that of sugar, and a flavor quite as agreeable and pleasant. The tablet form in which saccharin is now put up is very convenient for sweetening coffee, tea, and other beverages. Constant use of saccharin in practice for over a year has convinced me that it is entirely harmless in these cases.

The methed of dieting diabetic patients is of scarcely less importance than the quality of the diet itself. In order to more accurately determine the effects of diet upon the disease, no so-called specific medicines should be administered until the sugar excretion is reduced as far as is possible by diet alone. Step by step the more objectionable foods should be cut off until sugar ceases to appear in the urine, or until we reach almost—indeed in some cases an absolute—animal diet. Of course, where patients have been enjoying all the luxuries of a diet range comprising our modern resources of food supply and culinary art, an abrupt change to a strict diabetic diet would carry with it more or less danger, and therefore such course is never advisable. The *first step* should consist in the exclusion of potatoes sugar, and farinaceous foods, except leaving the patient the liberty of using a moderate amount of bread thinly cut and well-toasted on both sides. With these restrictions the patient should continue without other changes for about two weeks. In the milder cases this "first step" in dieting will have caused a reduction of the sugar in the urine to relatively small proportions; indeed, in some cases it completely vanishes. If sugar still appears in the urine—especially in considerable quantities—under the above restrictions, we may know that the disease is at least of moderately severe type, and we should proceed to the next step in diet. This should consist in the exclusion of milk, and all vegetables save green ones. Greater care should be exercised in the use of bread; white bread should be forbidden, and some substitute employed that contains less starch. Gluten or bran bread may be tried, but always toasted, as this alters its contained starch, so that it is not so readily converted into sugar.

After two weeks' adherence to the above restriction, if sugar still appears in the urine beyond mere traces, we may be sure that we have to deal with the disease in its more severe type, and we must accordingly bring to bear against it all our resources of diet in the most strict form. Everything containing starch, or sugar, that can be avoided, should be strictly forbidden. This last step should be entered upon rather more gradually than the others. Milk, if previously permitted, should now be replaced by pure cream. Cabbage, celery, radishes and string beans should be exchanged for spinach, lettuce, water-cresses, olives and cucumbers. Lastly, apples, tomatoes, and all fruits should be avoided, and with the exception of almond bread, some nuts and a few greens, the patient is reduced to an animal diet. Upon these restrictions, properly carried out, we shall find a large proportion of diabetic patients cease to excrete sugar with their urine, and with this result nearly all the symptoms of the disease will disappear.

In exceptional cases, even after a fair trial of the above restrictions, sugar still appears in the urine,but it rarely exceeds one per cent. Under such circumstances the patient should be placed upon an absolutely animal diet, at least for a time. It will be found that a strictly animal diet will often remove these last traces of sugar from the urine, and after its continuance for a longer or shorter time, a reversion to some of the less objectionable articles of the vegetable order causes no re-appearance of sugar in the urine.

In accustoming the patient to the more strict form of diet, care should be exercised not to permit the stomach to be overloaded. The beneficial effects of temperate eating in glycosuria were very prominently illustratcd during the siege of Paris, as Bouchard observed that sugar entirely disappeared from the urine of diabetics in whom up to the time it had persisted, even though they had been living on a carefully regulated diet. The diminution in the quantity of food, occasioned by its great scarcity during the siege, effected that which alteration in quality had failed to accomplish.

The more slowly food is submitted to the digestive forces, the more completely is it likely to become assimilated. Light meals frequently repeated is the better rule to follow, at least until the patient becomes more accustomed to the change. It is important also that the diet be varied as greatly from day to day as the range of food in the list will permit.

I have repeatedly placed diabetic patients that were considerably under twenty years of age upon the strict lines of diet herein indicated, with the result of completely eliminating the sugar from the urine for weeks and months together, and without resort to medication. Thus it may be seen how much may be expected from proper dieting, even in cases that we are forced to consider as ultimately hopeless ones.

By way of illustration—a year ago this month a lad of 18 years came to me from a distant State, with a history of diabetes of over a year's standing. His symtoms, as is usual in such cases, were great thirst, morbid appetite, polyuria, and advancing emaciation, with a very considerable amount of sugar in his urine. His physician at home had put him upon a diet scarcely so limited as the "first step" laid down in this paper, and but a slight check was put upon the disease. I gradually restricted his food allowance, until it conformed to the strict diabetic diet already laid down. His thirst gradually subsided, the quantity of urine diminished, and at the end of six weeks no trace of sugar was to be found in his urine, and he began to regain his lost weight. Under a continuance of this course the urine remained normal in quantity and free from sugar for about three months, when he returned to his home with directions to follow as closely as possible the course that had so greatly benefitted him. This case may be fairly ranged among the most unpromising ones, chiefly on account of the patient's age; for it is a rare exception to meet with a case under 20 years of age in which the disease does not rapidly prove fatal unless the patient be very strictly dieted.

It may be said of glycosuria in general that its severity is usually in inverse ratio to the age of the patient. The youngest diabetic patient I have seen came under my care a short time since, in the person of a little boy three years and two months old. In this case the polyuria was so pronounced that a nurse had to be provided to attend him at night, as he "wet the bed" from six to eight or more times each night. It may be of interest to note that he was put upon an animal diet, including milk, which soon lessened his polyuria so that the patient did not urinate during the whole night. I believe milk is more easily assimilated by children than by adults; at any rate it seems to agree better with them in these cases; and this is very fortunate, since we are almost driven to its use in diabetics of tender age. As a rule, in patients under middle age, we shall be obliged to bring to bear against glycosuria all our resources of dieting in the more strict form. I have met with an exception to this rule in the case of a Jewess, 29 years of age, in whom moderate restrictions of diet have kept the urine practically free from sugar for the past year and a half, only exceptional traces having appeared occasionally. It has been remarked by several observers that diabetes is frequent among Hebrews, and that in them the disease is always of mild form. My own experience tends to confirm the latter statement. I have, indeed, at the present time, three cases in Hebrew women under treatment, and they are all of mild form.

For the most part the milder forms of glycosuria are met with in people that have passed the age of 40 or 50 years. In this class of cases our resources against the disease are always mor effective; indeed, one or two years careful dieting not infrequently leads to permanent cure.

It remains to speak of the medicinal treatment of glycosuria, and I may as well state frankly at the beginning that I have little faith in the curative power of medication over the disease, while, on the contrary, I am satisfied that the use of drugs in these cases is often productive of harm. My conclusions upon this point have been reached through separating the dietetic from the medicinal treatment, and then comparing the results of each. When a system of diet and medication are employed together from the beginning, the benefits accruing from diet may be attributed to the medicines, while the unfavorable influence of medication may be attributed to the disease. Our faith has become so supreme in the efficiency of medication in these days, that we are apt both to permit ourselves to be misled in its favor, and to overlook its possible injurious effects.

Of the various drugs that have been recommended in glycosuria, opium, perhaps, maintains its reputation best and has become the most popular. Opium undoubtedly tends to restrain the excretion of sugar in these cases, but the doses necessary to accomplish this result are so large that the drug is likely to induce constipation and impaired digestion, and thus any good accomplished through its use is more than counterbalanced by resulting evil. I have recently gone over this ground very carefully in a series of trials systematically conducted. These cases were selected, in each of which the sugar excretion had been reduced by strict diet to about 1 per cent. They were all typical cases of true diabetes of central origin; and no little pains had been expended in reducing the sugar to so small a percentage, and maintaining a good general condition with excellent digestion and assimilation. Under gradually increasing doses of opium the sugar excretion was reduced somewhat in all the cases, but sooner or later constipation, loss of appetite, or nervous disturbances compelled the discontinuance of the drugs without exception. This has always been my experience in the use of opium in glycosuria, nor have I found any material advantage in the use of morphia, its bimeconate, or the use of cocaine. They all comport themselves much the same as does opium when used in equal physiological doses.

Ergot is probably the next most popular drug employed in the treatment of glycosuria. In the necessarily large doses required to effect the disease it is unsuitable for lengthy periods of administration. Its controling power over glycosuria is very feeble and uncertain, and on the whole it may be regarded as unworthy of much confidence.

Bromide of arsenic and syzgium jambolanum have recently been highly lauded in the treatment of glycosuria. I have known the former to be administered in the largest doses (25 drops Gilliford's solution), during which time the patient continued to excrete urine that contained 30 grains of sugar to the ounce. Upon withdrawing the bromide of arsenic and placing the patient upon a restricted diet, I had the satisfaction of seeing the sugar speedily reduced to 2 grains to the ounce. I have administered jambol to a number of my patients, but without noticing any favorable change that I could fairly ascribe to its use. A number of other drugs have been more or less highly extolled for their alleged specific influence over glycosuria. Among these may be mentioned iodoform, bromide of potassium, iodide of potassium, arsenic, sodium phosphate, nitrate of uranium, salicylic acid, picric acid and Calabar bean. There does not, however, appear to be sufficient evidence in favor of any one of these to entitle it to any degree of confidence. Carefully discriminated from the benefits derivable from dieting, these drugs are probably nearly inert, so far as their influence over glycosuria is concerned.

The legitimate field of therapeutics in glycosuria becomes practically narrowed down to the treatment of its accompanying symtoms, and upon this point but few words will be here added. It has already been stated that disordered digestion is so frequent in glycosuria as to constitute it an accompanying rule. Indeed, many of the milder cases owe their origin without doubt to this cause. The digestive and assimilative functions should therefore receive especial support through such agents as experience has taught us prove the most efficient. Among these may be mentioned, pepsin, and the vegetable bitters, and especially strychnia. The latter I have come to regard with increasing favor.

Constipation so frequent an accompaniment of glycosuria, should be especially guarded against, as this condition reacts very markedly in enfeebling the digestive and assimilative powers. I have an especial preference for the natural alkaline purgative waters to meet such requirements, since they relieve the over-acid condition of the intestinal canal so common to the disease. Friedrichshall or Sprudel—or the salt made by the evaporation of the latter—given before breakfast, in hot water, seem especially appropriate. In middle-aged people inclined to stoutness and over-eating, a course of purgation by either of these agents often proves highly beneficial.

The various nervous disturbances accompanying glycosuria are, on the whole, perhaps best met by the use of bromides, especially that of sodium or lithium. It is not uncommon to meet cases of glycosuria complicated by anæmia. When pronounced, this condition is frequently attended by œdema of the extremities, and under such circumstances the liberal use of iron and arsenic is attended by excellent results. The appearance of multiple boils is not uncommon in glycosuria patients; a complication generally considered ominous of approaching danger. I have seen a disappearance of this complication in two weeks under the use of quinine—8 to 10 grains daily—after having resisted other measures for nearly three months.

The most dangerous, and certainly the must rapidly fatal, of all the complications of glycosuria is that of Kussmaul's coma—sometimes called acetonæmia. Since the treatment of this complication has thus far proved so unsatisfactory, a knowledge of the conditions commonly leading thereto should be borne in mind, in order to guard the patient against it. Constipation, mental emotion, and fatigue seem especially to predispose to this complication, while a highly acid state of the urine often precedes it. I have repeatedly, in these cases, observed sudden death by coma to constitute the penalty of a hunting expedition, or long railway journey entailing unusual fatigue. If the early indications of approaching coma are observed, stimulants and hot baths should be resorted to without delay. It is believed that diabetic coma is brought about by some toxic agent in the blood, perhaps derived from alcoholic fermentation of glucose. Whether this be acetone, or some other agent, we are warranted by certain facts in believing that it is of an acid nature, and, therefore, large doses of alkalies seem the most appropriate remedies to employ. An ounce of tartrate or citrate of soda dissolved in a pint of water may be given three or four times a day. The intravenous injection of sodium carbonate, with chloride of sodium, is strongly advised if coma has already become established. Under the latter circumstances, however, recovery is extremely rare under any form of treatment. On the whole, then, promising results are only to be expected by attempts at warding off the attack through such measures as have already been suggested.

In concluding what has been intended as a practical review of the management of glycosuria, it seems desirable to emphasize the immense importance of careful dieting as greatly out-weighing all our other resources combined. This fact should be strongly impressed upon the patient from the beginning. He should be taught to rely little upon medication, and the most effective means of doing this is to show him how much can be accomplished by careful dieting alone. When he has once learned through experience that the amount of sugar in his urine always bears a direct ratio to the prohibited foods indulged in, he is less likely to overstep the proper limits imposed. With his thirst, polyuria, and other discomforts relieved—a sure sequence of careful conformances to the rules, unless he be greatly lacking in intelligence and gratitude, he will cheerfully submit to the conditions imposed, since he will see and feel how greatly he is indebted to them.

